# Identification and characterization of a novel molecular classification incorporating oxidative stress and metabolism-related genes for stomach adenocarcinoma in the framework of predictive, preventive, and personalized medicine

**DOI:** 10.3389/fendo.2023.1090906

**Published:** 2023-02-13

**Authors:** Ying Dong, Qihang Yuan, Jie Ren, Hanshuo Li, Hui Guo, Hewen Guan, Xueyan Jiang, Bing Qi, Rongkuan Li

**Affiliations:** ^1^ Gastroenterology and Hepatology Department, The Second Affiliated Hospital of Dalian Medical University, Dalian, China; ^2^ Department of Infectious Diseases, The Second Affiliated Hospital of Dalian Medical University, Dalian, China; ^3^ Graduate School of Dalian Medical University, Dalian Medical University, Dalian, China; ^4^ Department of General Surgery, The First Affiliated Hospital of Dalian Medical University, Dalian, China; ^5^ Department of Oncology, The First Affiliated Hospital of Dalian Medical University, Dalian, China; ^6^ Department of Dermatology, The First Affiliated Hospital of Dalian Medical University, Dalian, China

**Keywords:** oxidative stress, metabolism, stomach adenocarcinoma, predictive preventive personalized medicine (PPPM), molecular classification

## Abstract

**Background:**

Stomach adenocarcinoma (STAD) is one of the primary contributors to deaths that are due to cancer globally. At the moment, STAD does not have any universally acknowledged biological markers, and its predictive, preventive, and personalized medicine (PPPM) remains sufficient. Oxidative stress can promote cancer by increasing mutagenicity, genomic instability, cell survival, proliferation, and stress resistance pathways. As a direct and indirect result of oncogenic mutations, cancer depends on cellular metabolic reprogramming. However, their roles in STAD remain unclear.

**Method:**

743 STAD samples from GEO and TCGA platforms were selected. Oxidative stress and metabolism-related genes (OMRGs) were acquired from the GeneCard Database. A pan-cancer analysis of 22 OMRGs was first performed. We categorized STAD samples by OMRG mRNA levels. Additionally, we explored the link between oxidative metabolism scores and prognosis, immune checkpoints, immune cell infiltration, and sensitivity to targeted drugs. A series of bioinformatics technologies were employed to further construct the OMRG-based prognostic model and clinical-associated nomogram.

**Results:**

We identified 22 OMRGs that could evaluate the prognoses of patients with STAD. Pan-cancer analysis concluded and highlighted the crucial part of OMRGs in the appearance and development of STAD. Subsequently, 743 STAD samples were categorized into three clusters with the enrichment scores being C2 (upregulated) > C3 (normal) > C1 (downregulated). Patients in C2 had the lowest OS rate, while C1 had the opposite. Oxidative metabolic score significantly correlates with immune cells and immune checkpoints. Drug sensitivity results reveal that a more tailored treatment can be designed based on OMRG. The OMRG-based molecular signature and clinical nomogram have good accuracy for predicting the adverse events of patients with STAD. Both transcriptional and translational levels of ANXA5, APOD, and SLC25A15 exhibited significantly higher in STAD samples.

**Conclusion:**

The OMRG clusters and risk model accurately predicted prognosis and personalized medicine. Based on this model, high-risk patients might be identified in the early stage so that they can receive specialized care and preventative measures, and choose targeted drug beneficiaries to deliver individualized medical services. Our results showed oxidative metabolism in STAD and led to a new route for improving PPPM for STAD.

## 1 Introduction

Stomach adenocarcinoma (STAD) is the most frequent histological form of gastric cancer (GC), which is ranked the third major contributor to cancer-caused deaths (1). Nearly half (47%) of the world’s new GC cases are diagnosed in China annually; over 60% of these patients experienced advanced disease at the time of diagnosis and treatment with a 5-year survival rate below 30% (2, 3). Stomach cancer responds poorly to standard therapies including surgery, chemotherapy, and radiotherapy. Even though molecularly targeted drugs have been developed for stomach cancer, the targeted treatment for this illness is still years behind what it is for lung cancer, breast cancer, colon cancer, and other common forms of cancer (4, 5). As a result, it is of the utmost importance to locate valid biomarkers to accurately anticipate the outcome (prognosis) of STAD patients and to provide individualized therapy.

Oxidative stress is characterized by increased levels of intracellular reactive oxygen species (ROS), which may be harmful to DNA as well as proteins and lipids (6). According to evidence, excessive ROS production results in oxidative stress in tissues and cells, which results in several disorders, including cancer (7, 8). ROS is a potent mutagen that has been linked to the onset and advancement of cancer (9). Meanwhile, oxidative stress promotes tumor cell survival and develops tumor angiogenesis and metastasis (10, 11). High-speed cell development in solid tumors causes insufficient blood flow, which creates hypoxic areas within the tumor and encourages the creation of ROS (12). Metabolic reprogramming is a feature that is common in many different types of solid tumors and affects the availability of nutrients and the manner that cells utilize those resources to fulfill the material and energy needs of cancer (13–15). Oncogene-driven metabolic modifications provide cancerous cells with the ability to survive and thrive in the tumor microenvironment (TME) (16). The prognosis of patients and their responsiveness to chemotherapy and immunotherapy are strongly correlated with metabolic heterogeneity (17, 18). Previous research has shown that patients with GC have considerable metabolic remodeling (19, 20). As per our current understanding, the prognostic capabilities of oxidative stress and metabolism in GC are yet to be clarified. In-depth research on oxidative stress and metabolism has not yet been conducted to determine how these factors influence GC in a global manner. As a result, additional research must be conducted into the relationship between oxidative stress and metabolism-related genes (OMRGs) and GC.

In this study, we evaluated the copy number variation (CNV), single nucleotide variation (SNV), methylation, mRNA expression, prognostic significance, and pathway regulation changes in the OMRGs of various cancer types using gene data and clinical data gleaned from The Cancer Genome Atlas (TCGA) database. Then, by using bioinformatics techniques, we comprehensively assessed the OMRGs and STAD prognosis. As per the total oxidative metabolism score as well as the expression levels of OMRGs, we classified the dataset of STAD patients into three clusters. We also examined how these three clusters were connected to prognosis and therapies for patients. Owing to the mounting evidence suggesting a pivotal function for immune cells and immune checkpoint genes (ICGs) in tumorigenesis (21, 22), We probed whether the oxidative metabolism score was linked to ICI and ICGs. Additionally, we developed a risk score model based on13 OMRGs to anticipate the prognosis of STAD patients. Finally, the development of a nomogram to forecast survival probabilities for patients with STAD could be employed to bolster clinical judgment and tailor treatment for each patient. Our findings provided promising insights into oxidative stress and metabolism in STAD and paved the way for directing clinical therapy for individualized treatment within the framework of predictive, preventive, and personalized medicine (PPPM).

## 2 Materials and methods

### 2.1 Data collection and processing

The TCGA database was searched to retrieve RNA-sequencing (RNA-seq) data and matched clinical features from the TCGA-STAD cohort. The GSE84437 cohort’s RNA-seq and clinical data were acquired from the GEO database. Following the exclusion of patients with a survival of 30 days or less, bioinformatics analysis was performed on 312 STAD and 32 normal samples from TCGA, and 431 STAD samples from GSE84437 (23). Batch normalization was done with the aid of the “sva” package in R (24). We also obtained SNV data, transcriptome profiles, CNV data, methylation data, and clinical variables of pan-cancer transcriptomes from the TCGA platform (25).

In addition, 3098 oxidative stress-related genes and 2803 metabolism-related genes (Relevance score>2.5) were acquired from the GeneCard Database (https://www.genecards.org/). GeneCards mines and integrates comprehensive information on human genetics from more than 80 data sources and provides concise genome, proteome, transcriptome, disease, and function data on all known and predicted human genes. After taking the intersection of the two groups of genes, 1520 OMRGs were obtained and displayed by a Venn diagram. The OMRGs in the TCGA and GEO cohorts with prognostic significance were obtained *via* univariate cox regression analysis. Finally, we obtained 22 OMRGs with prognostic significance and applied the “corrplot” package to visualize the co-expression correlation between any two OMRGs. The prognostic significance of 22 OMRGs was also validated using Kaplan-Meier (KM) analysis based on 743 STAD samples.

### 2.2 Pan-cancer analysis

Currently, despite the fact that there may be a link between oxidative metabolism and cancer, very little research has been performed on the topic. Therefore, there is a lack of a comprehensive account of how OMRGs vary between cancer types. To offer a holistic view of OMRGs across cancer types, we examined and visually displayed data on SNVs, CNVs, methylation, and mRNA expression. To additionally evaluate the roles of OMRGs in cancer prognosis, we examined the link between mRNA expression and OS by a univariate Cox regression model. The R programming language was applied to perform all analyses (26, 27).

To uncover the unique role of pathways affected by OMRGs across a spectrum of human cancers, we employed single-sample gene set enrichment analysis (ssGSEA) to calculate OMRGs scores in each cancer sample. Both the top and bottom 30% of OMRG scores were used to classify the samples into two groups. Differences (variations) in pathway activity across the two groups premised on the transcriptomes were studied *via* gene set enrichment analysis (GSEA).

### 2.3 Analysis of clusters premised on oxidative metabolism score

We generated an oxidative metabolism score model as per mRNA expression to highlight the differential expression levels across the samples as a result of the huge discrepancies in gene expression patterns that were seen in the previously collected datasets. In short, the enrichment scores of OMRGs were initially assessed using a ssGSEA (28). The RStudio “Gplots” package was utilized for performing the differential analysis, while the “pheatmap” program was employed to generate the heat map of the cluster analysis outcomes. Comparing the mRNA expression patterns of the genes in normal samples with those in cancer tissues allowed for the classification of the tumors into three categories (groups) based on their mRNA expression status: oxidative metabolism inactive (cluster 1 or C1), oxidative metabolism active (cluster 2 or C2), and normal oxidative metabolism (cluster 3 or C3). To additionally emphasize the links between the gene expression profiles of these 3 clusters, we employed the violin plot to illustrate the enrichment scores of the clusters, which were generated with the aid of the “ggpubr” package (29). Afterward, Kaplan-Meier (KM) analysis was executed to evaluate the prognostic relevance of clusters. Finally, we downloaded and curated 50 typical hallmark pathways from the Molecular Signatures Database (MsigDB) (30). Through ssGSEA analysis of tumor cells and normal cells in each sample, we obtained the enrichment score of each pathway. A heatmap was utilized to show the discrepancies between pathway enrichment scores and oxidative metabolism scores among three clusters. P< 0.05 signifies statistical significance.

### 2.4 Association between the oxidative metabolism score and immune status

As part of the ESTIMATE (Estimation of Stromal and Immune cells in Malignant Tumor tissues using Expression data) study, the immune and stroma scores were derived utilizing the “estimate” in the R program. In addition, the algorithm enabled the assessment of tumor purity. The 29 TCGA-retrieved immune-associated gene sets were quantified using ssGSEA, yielding 707 genes in total that reflect distinct immune cell types, functions, and pathways (31–33). ssGSEA allows for the study of gene signals produced by multiple cell groups within the immune system in a single sample. To assess and visualize the link between the oxidative metabolism score and immunological components, we utilized the RStudio packages “ggstatsplot,” “data.table,” “dplyr,” “tidyr,” and “ggplot2.” The area of each sphere in the mapped figure stands for the degree of correlation, whereas the color stands for the p-value. Furthermore, the “ggscatterstats” program was employed to create a scatter plot showing the relationships among the six standard immune cell groups (viz., Macrophages, CCR, mast cells, type II interferon (IFN) response, Dendritic cells (DCs), and T follicular helper (Tfh) cells), and the oxidative metabolism score. In addition, When comparing the levels of expression of common ICGs across low- and high-risk groupings, we only displayed statistically significant findings. p< 0.05: statistical significance.

### 2.5 Drug sensitivity analysis

Each STAD patient’s medication responsiveness was predicted utilizing the “pRRophetic” R package (34, 35), and the possibly sensitive medications were screened for high- and low-risk categories with the “Wilcox.test” function in R. It should be noted that drug sensitivity improves with decreasing IC50 values. p< 0.05:statistical significance.

### 2.6 Construction and verification of a prognostic signature based on OMRGs

Then, LASSO-Cox regression analysis was applied to 22 OMRGs linked to patients’ prognoses, with minimal criteria determining the penalty parameter (λ) (36). The equation for computing the risk score was as follows: Risk score = $\sum_{k=1}^{n}expk*\beta k$ . We used the median risk score to designate STAD patients in the TCGA and GSE84437 cohorts as either high- or low-risk. For further analysis, the TCGA cohort served as the training cohort, whereas the GSE84437 cohort served as the test cohort.

For the formulation and verification of the signature, the following stages were performed on the train and test cohorts (1): visualizing sample categorization was done using principal component analysis (PCA) (2); a survival study using the KM technique was carried out to determine whether the signature could be used to forecast survival (3); time-dependent receiver operating characteristic (ROC) curves and AUC values were created with the aid of the “survival ROC” R package to evaluate the sensitivity and specificity of the risk score.

### 2.7 Variations in ICG expression and immune function between low- and high-risk populations

First, we evaluated the variations in the expression of common ICGs between high- and low-risk groups, and we only showed findings that were statistically significant (p< 0.05). The correlation between immune function and risk levels was then investigated. The above analysis was performed in both the train and test cohorts.

### 2.8 Creating a predictive nomogram that incorporates clinical characteristics and risk score

Each patient’s clinical information (age, gender, grade, and stage) was retrieved from TCGA cohorts along with their risk scores. The indicators that showed statistical significance (p< 0.05) in the univariate Cox survival analysis were subsequently incorporated into the multivariate Cox survival analysis. These indicators independently functioned as prognostic variables as per the results of the multivariate analysis (p<0.05). The aforementioned clinical characteristics and risk score were used to develop a nomogram. Thereafter, the nomogram’s discriminating power and prediction accuracy were assessed using calibration curves. The prediction performance was also assessed using the time-dependent ROC curve.

### 2.9 Verification of thirteen model genes by GEPIA and HPA platforms

GEPIA is a web-based data management system for the systematic study of enormous volumes of RNA-seq data from the TCGA and GTEx datasets (http://gepia.cancer-pku.cn/) (37). We used the GEPIA database to compare the expression of 13 OMRGs between cancer and paired normal tissues. The Human Protein Atlas (https://www.proteinatlas.org/) is a database of immunohistochemistry-based protein expression profles in cancer tissues, normal tissues, and cell lines (38). We used it to compare the protein expression levels of 13 OMRGs between cancer and normal samples. However, CTLA4 and GRP protein expression data could not be located in the HPA database, thus we reported the protein expression levels of the remaining 11 OMRGs. In addition, antibody staining in various forms of cancer found in human tissue was classified as not observed, low, medium, or high in the HPA dataset. This score was computed by considering both the intensity of staining and the proportion of total stained cells. Similarly, the HPA database was utilized to demonstrate the immunofluorescence localization of cells.

## 3 Results

### 3.1 Data procession

**Figure 1:**
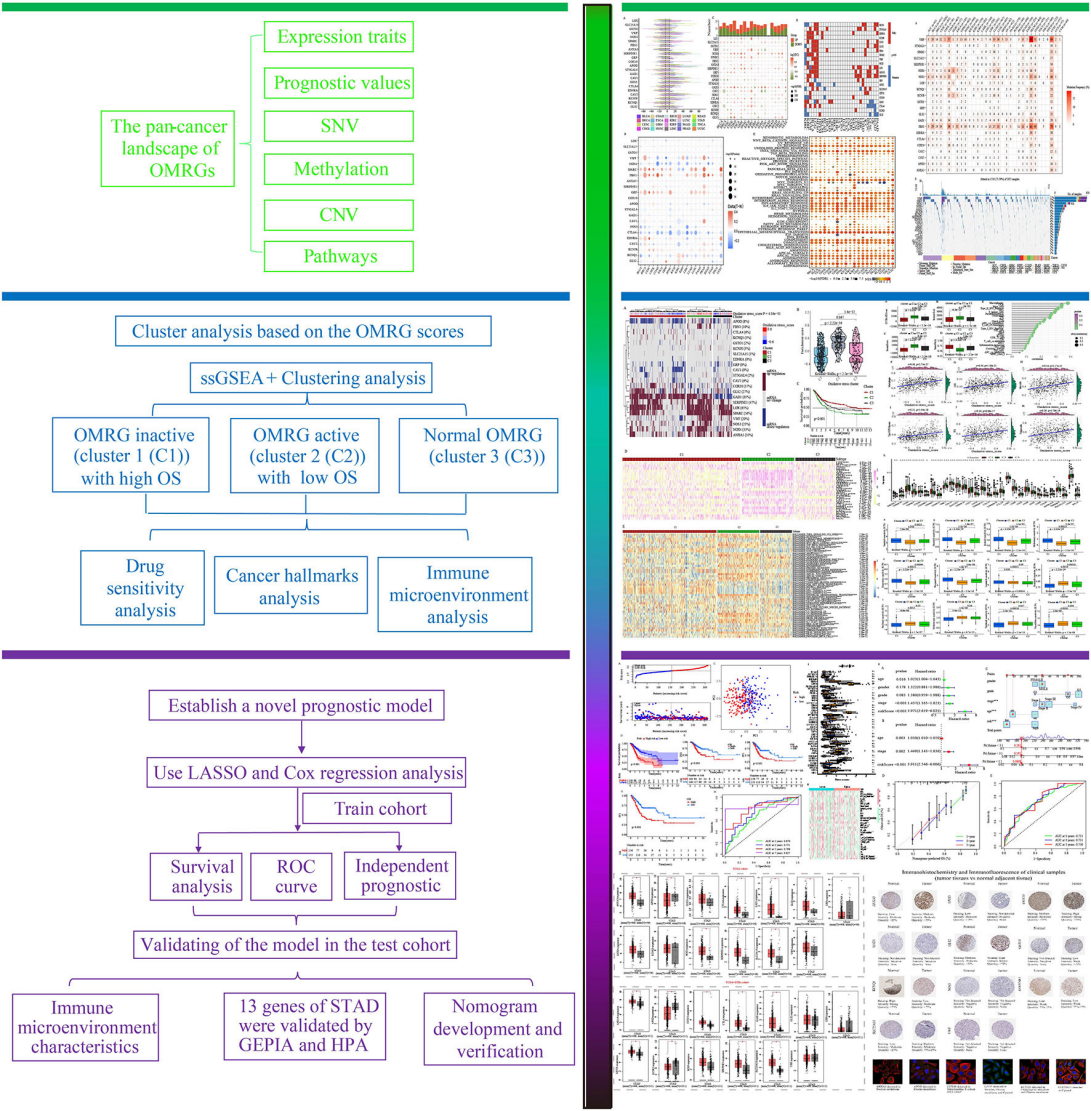
The flowchart of the research methodology.

**Figure 2:**
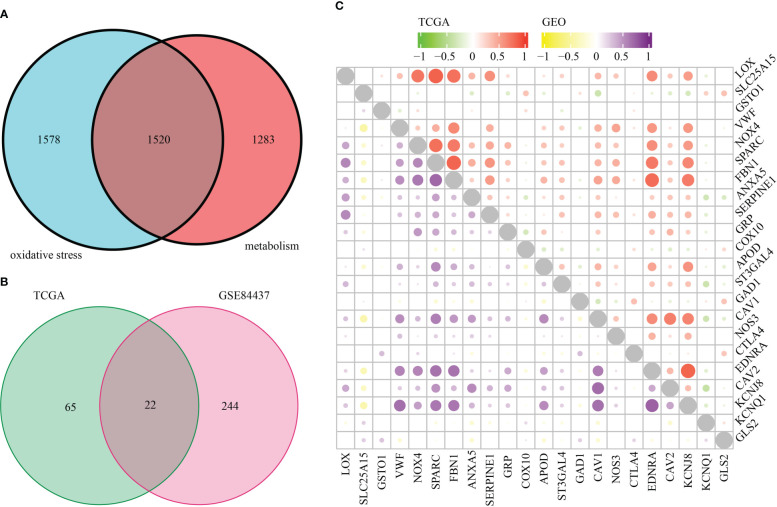
22 oxidative metabolism-related genes (OMRGs) with prognostic significance were obtained for subsequent analysis. **(A)** Venn diagram to find 1520 OMRGs. **(B)** Venn diagram depicting 22 prognostic OMRGs in both TCGA and GEO datasets. **(C)** The plot shows the result of co-expression relationships of 22 OMRGs in STAD. The size of the dot reflects the value of the p.

**Figure 1** is a flowchart that demonstrates the processes involved in the present research. First, we took the intersection of oxidative stress-related genes and metabolic-related genes to obtain 1520 OMRGs (**Figure 2A**). Then, the intersection was taken after univariate analysis in TCGA and GEO cohorts respectively, and 22 OMRGs with prognostic significance were obtained for analysis in this study (**Figure 2B**). KM analysis based on 743 STAD samples validated the prognostic significance of 22 OMRGs (**Supplementary Figure 1**). To explore the relationship between OMRGs, a co-expression analysis of the 22 OMRGs was carried out. As per the findings, the majority of genes had a positive link to one another across both the TCGA and the GEO cohorts. However, SLC25A15 was negatively associated with most genes (**Figure 2C**).

#### 3.1.1 Pan-cancer analysis of OMRGs

**Figure 3:**
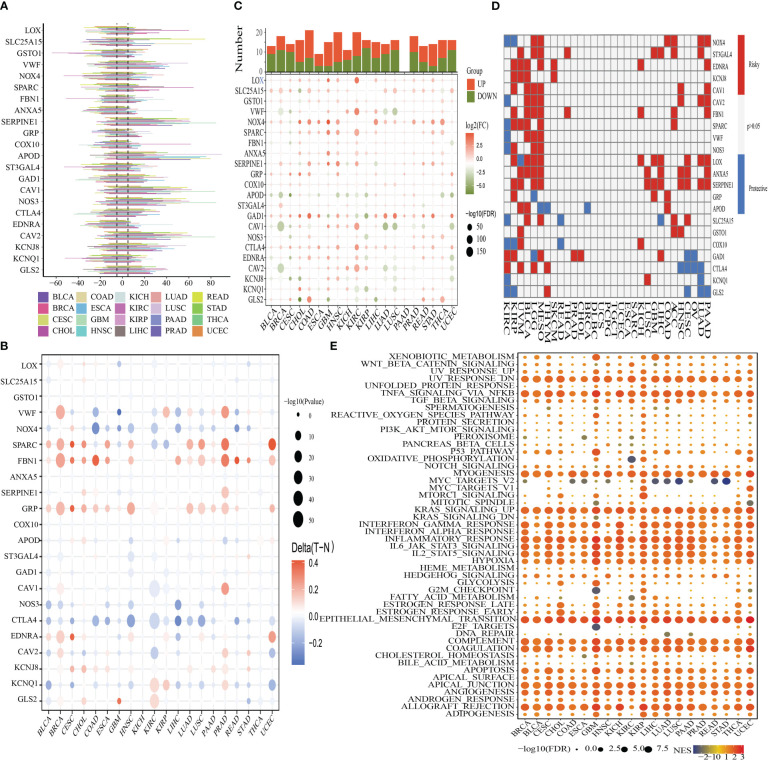
Panoramic depiction of OMRGs in pan-cancer. **(A)** The frequency of copy number variation (CNV) for each OMRG in each type of cancer is displayed in a histogram. **(B)** Genes that are hypermethylated and hypomethylated are indicated in red and blue, respectively, on a heatmap that displays the differential methylation of OMRGs in malignancies (Wilcoxon rank-sum test). **(C)** The histogram in the top panel shows the total number of substantially differentially expressed genes, whereas the heatmap depicts the fold change and FDR of OMRGs in each tumor. **(D)** OMRGs’ survival profiles across cancers. **(E)** Comparison of tumor samples with high and low OMRGs scores by performing enrichment analysis for cancer pathway signaling.

The CNV, methylation, SNV, mRNA expression patterns, and survival data for 22 OMRGs across a diverse range of cancer types for the pan-cancer investigation were derived from TCGA. To identify the frequency and types of variants in each cancer subtype, we evaluated SNV data linked to OMRGs. **Supplementary Figure 2A** demonstrates that SKCM, UCEC, LUSC, and COAD all displayed remarkable SNV frequency of OMRGs. The OMRGs have a 73.59% (1510 of 2052 tumors) frequency of SNVs. Based on the results of the variant analysis, missense mutations were shown to be the most common form of SNP. As per the percentage of SNVs, the five most mutated genes were identified as follows: VWF, FBN1, NOS3, NOX4, and GAD1, of which the mutation percentages were 22%, 22%, 10%, 8%, and 7%, respectively (**Supplementary Figure 2B**). To delve deeper into the genetic alterations of OMRGs that occur in cancer, the proportion of CNV was analyzed. The findings revealed that while general CNV appeared at high frequencies in the majority of cancers (**Figure 3A**), all of the OMRGs in THCA demonstrated a low frequency of CNV. OMRGs displayed a wide variety of CNV characteristics. For instance, SERPINE1, APOD, CAV1, NOS3, and CAV2 were more likely to achieve CNV gain than a loss in almost all cancers, but GSTO1 and ST3GAL4 had the reverse profile. In addition to CNV, promoter methylation can control gene expression, and abnormal promoter DNA methylation is linked to cancer (39). We noticed that among the 20 cancer types, the majority of OMRGs displayed intricate methylation patterns. However, FBN1 and GRP consistently showed hypermethylation in several tumors, while NOS3 and CTLA4 showed the opposite (**Figure 3B**).

For every cancer type, differential expression analysis was done to evaluate the variations in the gene expression patterns of OMRGs in addition to genetic changes between the tumor and nearby normal tissues. The findings illustrated that most of the gene expression profiles in cancer tissues were distinct from those observed in healthy tissues, except for those found in pancreatic cancer tissues. Significantly elevated expression profiles of LOX and NOX4 were seen in a variety of malignancies (**Figure 3C**). Subsequently, as depicted in **Figure 3D**, we applied univariate Cox regression of mRNA expression and OS to discover risky OMRGs with HR > 1 and p-value of< 0.05 as well as protective OMRGs with HR< 1 and p-Value of< 0.05. We found that most of the genes were risk factors in several cancers, except for CTLA4, KCNQ1, and GLS2. Since it is currently unknown how oxidative metabolism regulates pathways connected to cancer, it is imperative to examine any possible connections between these pathways and OMRGs. This will establish the groundwork for future research into how OMRGs regulate pathways related to pan-cancer. We discovered that TNFA signaling through NFKB, KRAS signaling, epithelial-mesenchymal transition (EMT), the inflammatory response, hypoxia, and the interferon-gamma response, were all remarkably correlated with OMRGs in pan-cancer (**Figure 3E**).

### 3.2 Cluster analysis centered on the oxidative metabolism score

**Figure 4:**
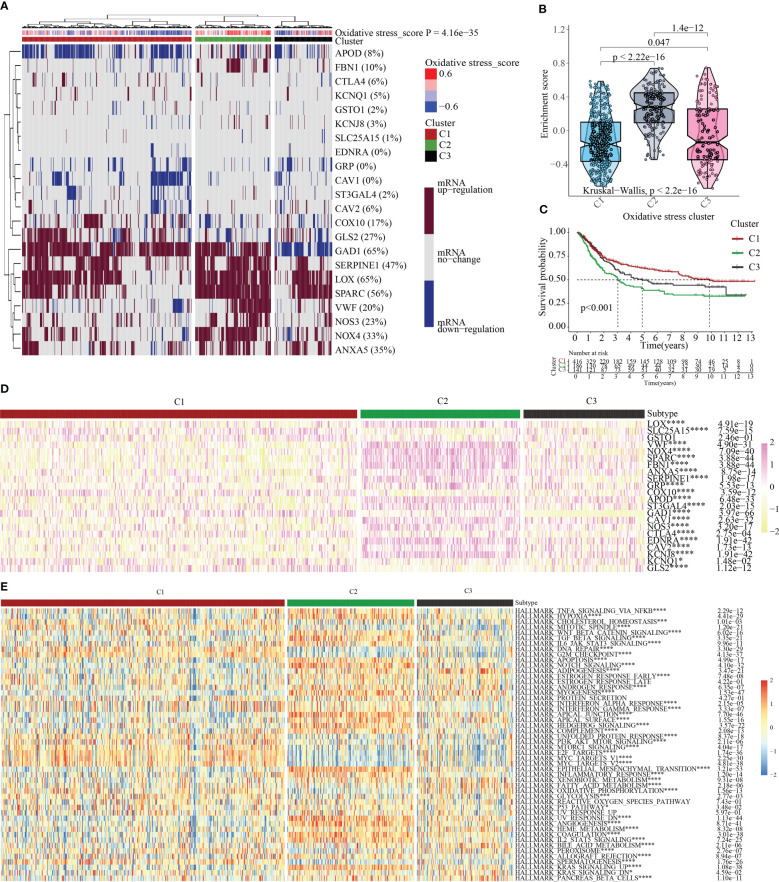
Oxidative metabolism scores-based cluster analysis. **(A)** Heat map cluster analysis of TCGA and GEO gene data illustrating three distinct clusters: inactive oxidative metabolism (cluster 1/C1), active oxidative metabolism (cluster 2/C2), and normal oxidative metabolism (cluster 3/C3). **(B)** C2 has the highest enrichment score, followed by C3 and C1 in descending order, as observed by the “ggpubr” package’s violin plot. **(C)** The survival curve of the three clusters. Years (depicted by the abscissa) are plotted against a survival rate (denoted by the ordinate). **(D)** The results of differentially expressed OMRGs between three clusters. **(E)** The relationship between cancer signaling and OMRGs (red color to blue color signifies high to low). *P < 0.05; ***P < 0.001; ****P < 0.0001.

We clustered the OMRGs and separated the 743 STAD patients into three categories predicated on their final oxidative metabolism score and gene expression patterns for the purpose of delving deeper into the connection between oxidative metabolism and STAD. C1 contained tumor tissues with inactive oxidative metabolism, C2 featured tumor tissues with active oxidative metabolism, and C3 contained tumor tissues with normal oxidative metabolism (**Figure 4A**). As per the violin plot, the following is the sequence in which the enrichment scores for the 3 clusters appeared: C2 > C3 > C1 (**Figure 4B**). The survival curves for the three groupings were then plotted to see if the clustering made sense. Patients in group C2 had the highest OS while those in group C1 had the opposite (**Figure 4C**), indicating that the oxidative metabolism score represents a risky factor. The heatmap illustrated the expression profiles of 22 OMRGs in the three subgroups (**Figure 4D**). Finally, the links between cancer hallmarks and oxidative metabolism scores were evaluated, and the findings revealed that more than half of the hallmarks were frequently remarkably linked to oxidative metabolism scores (**Figure 4E**).

### 3.3 Correlations between the oxidative metabolism score and ICI

**Figure 5:**
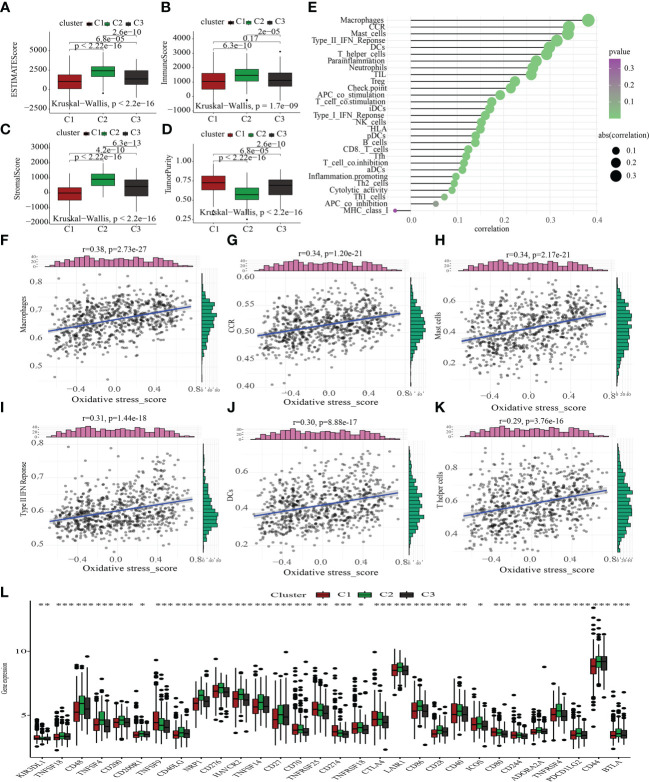
Comparative immunological status examination of two molecular groups. **(A-D)** Comparative analysis of the TME components. **(E)** The plot illustrates the link between the score for oxidative metabolism and the infiltration of immune cells. **(F-K)** The scatter figure illustrates the link between the oxidative metabolism score and six substances associated with immune infiltration. A favorable correlation was identified between the oxidative metabolism score and the infiltration of macrophages, CCR, mast cells, type II IFN response, DCs, and T helper cells. **(L)** Comparison of the three subtypes’ immune checkpoint gene expression using a differential analysis method. *P < 0.05; **P < 0.01; ***P < 0.001.

The tumor microenvironment (TME) encompasses stromal, tumor, and immune cells, as well as secreted chemokines and cytokines (40), which modulate the onset and advancement of cancer (41). The clinical outcome of cancer is tightly tied to immune cells, which are an important part of the TME and useful anticancer therapeutic targets (42). We evaluated TME components in C1, C2, and C3 to probe into the link between oxidative metabolism and immune response among STAD patients. The TME components in the different oxidative metabolic clusters were as follows: ESTIMATEScore: C1 > C3 > C2; ImmuneScore: C1 > C3 > C2; StromalScore: C1 > C3 > C2; tumor purity: C2 > C3 > C1 (**Figures 5A–D**). Next, we examined how the oxidative metabolic score interacted with ICI (**Figures 5E–K**) and discovered that it was positively linked to the infiltration levels of macrophages, CCR, mast cells, type II IFN response, DCs, and T helper cells. As a compensatory strategy, a higher incidence of ICI may have occurred due to a deficient local immune response. As shown in **Figure 5L**, most ICGs expression was enhanced in the C2 subtype. Increased ICG expression inhibited effective anti-cancer immune responses, thereby inducing the migration of immunocytes into the TME to enhance compensatory responses.

### 3.4 Association of drug sensitivity with the oxidative metabolism clusters

**Figure 6:**
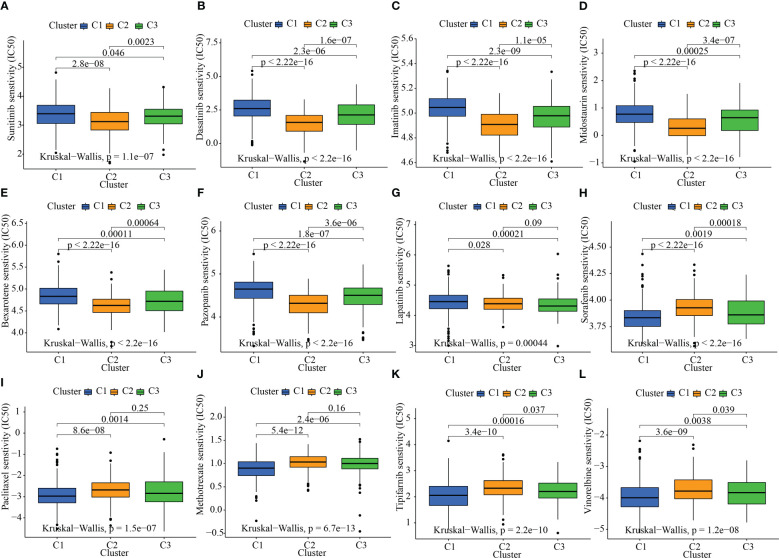
The link between sensitivity to pharmaceuticals and clusters of oxidative metabolism. The box plots of the predicted IC50 values for twelve different kinds of commonly used chemotherapy drugs are displayed in **(A–L)** for cluster 1 (blue), cluster 2 (yellow), and cluster 3 (green). The following are the 12 different kinds of chemotherapy-related drugs: Sunitinib, Dasatinib, Imatinib, Midostaurin, Bexarotene, Pazopanib, Lapatinib, Sorafenib, Paclitaxel, Methotrexate, Tipifarnib, and Vinorelbine.

Given that molecularly targeted treatment is used to treat STAD at present, we tested these 3 oxidative metabolism clusters against 12 different medications. The majority of these drugs are either frequently used targeted therapies, especially STAD, or standard pharmacological treatments utilized in the study of tumors. As per the results of the ridge regression model, the various angiogenesis clusters showed the following patterns of drug sensitivity: Sunitinib: C2 > C3 > C1; Dasatinib: C2 > C3 > C1; Imatinib: C2 > C3 > C1; Midostaurin: C2 > C3 > C1; Bexarotene: C2 > C3 > C1; Pazopanib: C2 > C3 > C1; Lapatinib: C3 > C2 > C1; Sorafenib: C1 > C3 > C2; Paclitaxel: C1 > C3 > C2; Methotrexate: C1 > C3 > C2; Tipifarnib: C1 > C3 > C2; and Vinorelbine: C1 > C3 > C2 (**Figures 6A–L**).

### 3.5 Determination and verification of an OMRGs-based prognostic signature

**Figure 7:**
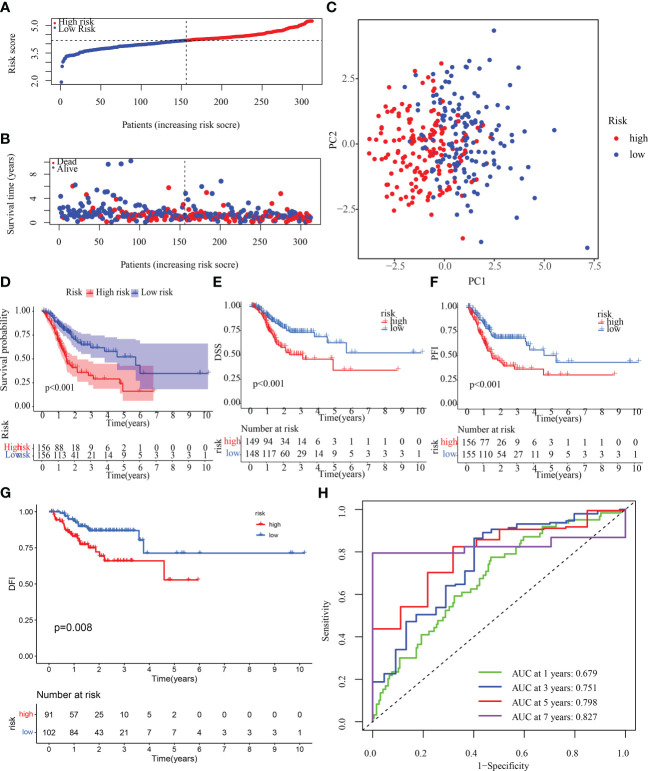
Construction of OMRG-related signature in the training cohort. **(A)** Various groups of the training cohort were created premised on the median risk score. **(B)** The survival rate of the training cohort as well as the distributions of risk scores **(C)** PCA for the training cohort. **(D-G)** KM analyses (OS, DSS, DFI, and PFI) of the training cohort. **(H)** Values of the AUC for the training cohort.

To determine if the OMRGs might be used to generate a signature for anticipating the therapeutic outcomes of STAD patients, LASSO-Cox regression was applied to analyze the 22 genes. Ultimately, 13 genes were chosen to create the risk score model (**Supplementary Figure 3**). Risk score = (-0.054980970832357) * expression of SLC25A15 + 0.332441495029009 * expression of GSTO1 + 0.0711757410696192 * expression of VWF+ 0.08897393300098 * expression of ANXA5 + 0.144637581187946 * expression of SERPINE1 + 0.140239358545706 * expression of GRP + (-0.0683192836732951) * expression of COX10 + 0.0057794428801345 * expression of APOD + (-0.104333942341152) * expression of GAD1 + 0.140768401184509 * expression of NOS3 + (-0.19273718582153) * expression of CTLA4 + (-0.0301338387672) * expression of KCNQ1 + (-0.0404719679140591) * expression of GLS2. Then, samples from the training cohort were stratified into high- and low-risk categories premised on the median risk score (**Figure 7A**). Patients who had high-risk scores exhibited a dismal chance of survival, as measured by the probability distributions of risk scores (**Figure 7B**). **Figure 7C** shows a PCA that can be used to easily differentiate between high- and low-risk groups (categories) The high-risk category exhibited a substantially more unfavorable OS, disease-specific survival (DSS), disease-free interval (DFI), and progression-free interval (PFI) (p< 0.05), as depicted in **Figures 7D-G**. In addition, the 1-, 3-, 5-, and 7-year AUC values for the survival rate of the ROC curves of risk score were 0.679, 0.751, 0.798, and 0.827, correspondingly (**Figure 7H**), indicating that the risk score is a significant factor in the survival prediction of patients with STAD. In addition, to further verify the accuracy of the signatures, we compared our signatures with seven other published signatures (43–49). C-index and ROC curves illustrated that our signature prediction ability was significantly better than the other seven published signatures (**Supplementary Figures 4A-I**).

### 3.6 Verifying the prediction value of the risk signature in the test population

**Figure 8:**
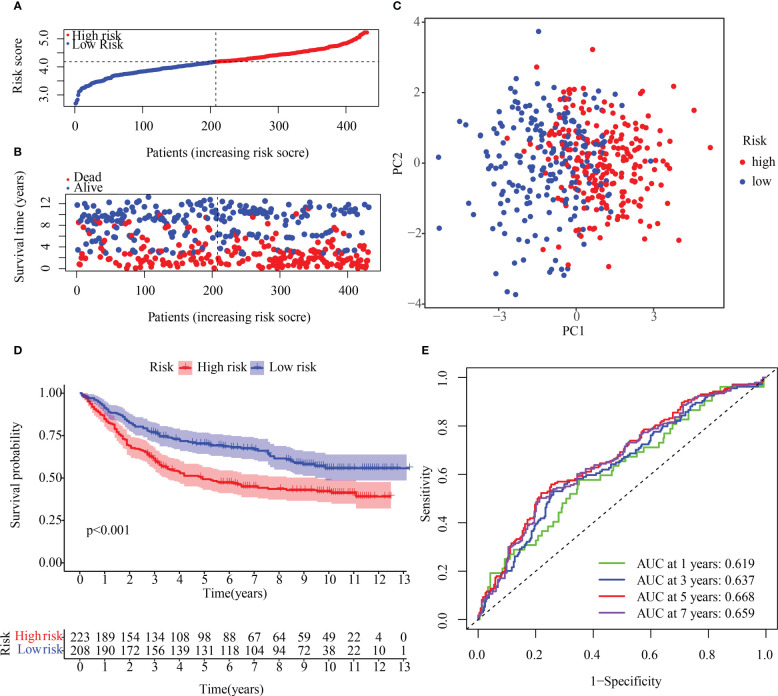
Internal validation of the OMRG-related signature in the test cohort **(A)** The test cohort was subdivided into various subgroups. **(B)** The survival rate of the test cohort as well as the distribution of risk scores **(C)** Principal component analysis was performed on the test1 cohort. **(D)** Survival curve of the testing cohort. **(E)** AUC values of ROC curves in the test cohort.

To verify the reproducibility of the risk score in another patient population, OMRG expression was measured in 431 HCC patients with complete survival data from the GEO cohort (GSE84437). The train cohort’s median risk score was used to classify the GEO dataset into high- and low-risk subsets (**Figure 8A**). **Figure 8B** shows that the low-risk population had a higher survival rate relative to the high-risk category, which had more deaths overall. The PCA method was used to further classify patients in the two risk categories into two groups (**Figure 8C**). KM curves for OS depicted in **Figure 8D** illustrate that the high-risk patients had a poorer prognosis in contrast with those with low risk, with the high-risk population experiencing a shorter OS duration. A time-dependent ROC curve was examined to further gauge the predictive risk signature’s accuracy. The risk score’s outstanding diagnostic utility was demonstrated by the AUC of its ROC curves (**Figure 8E**).

### 3.7 ICG expression and immune function differences between low- and high-risk categories

**Figure 9:**
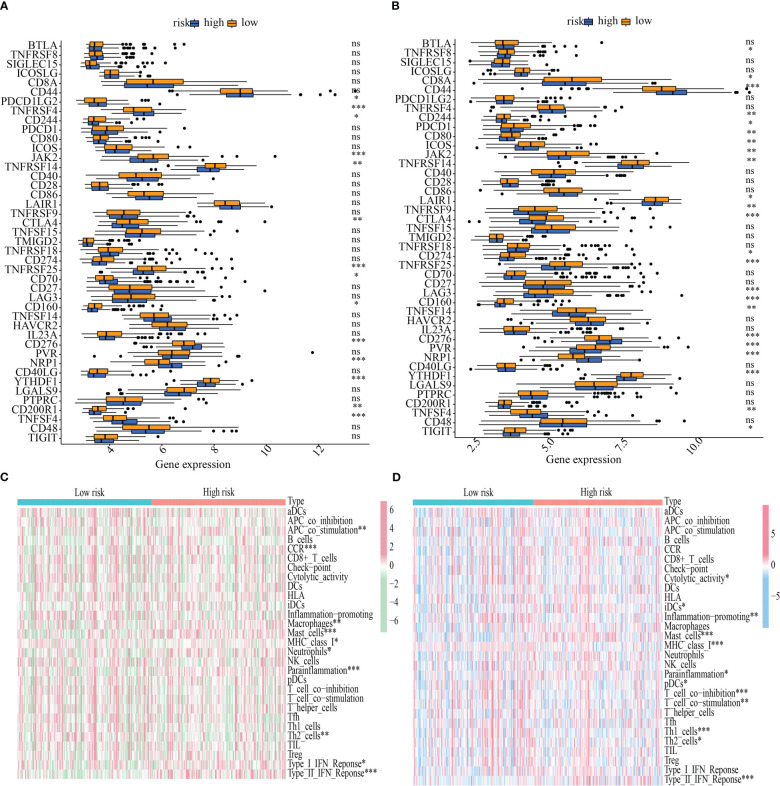
Immunological function abundance and immune checkpoint gene expression variations in groups with low and high risk. **(A-B)** Analysis of the differential expression of ICGs between the training and testing cohorts. **(C-D)** The state of immune function across the training and the testing cohorts. *P < 0.05; **P < 0.01; ***P < 0.001. ns, no significance.

The influence that varying levels of ICG expression had on the tumor immune milieu of the tumor was studied. In both the training and testing cohorts, YTHDF1, CD160, TNFRSF25, CTLA4, TNFRSF14, JAK2, and CD244 were more highly expressed in the low-risk subgroup, whereas TNFSF4, NRP1, CD276, and CD244 were overexpressed in the high-risk population (**Figures 9A, B**). Also, a heatmap was generated to ascertain the link between the risk score and immune function. Mast cell function, MHC class I expression, parainflammation, Th2 cells, and Type II IFN response all varied substantially between the high- and low-risk categories in both the training and testing sets (**Figures 9C, D**).

### 3.8 Nomogram development and verification

**Figure 10:**
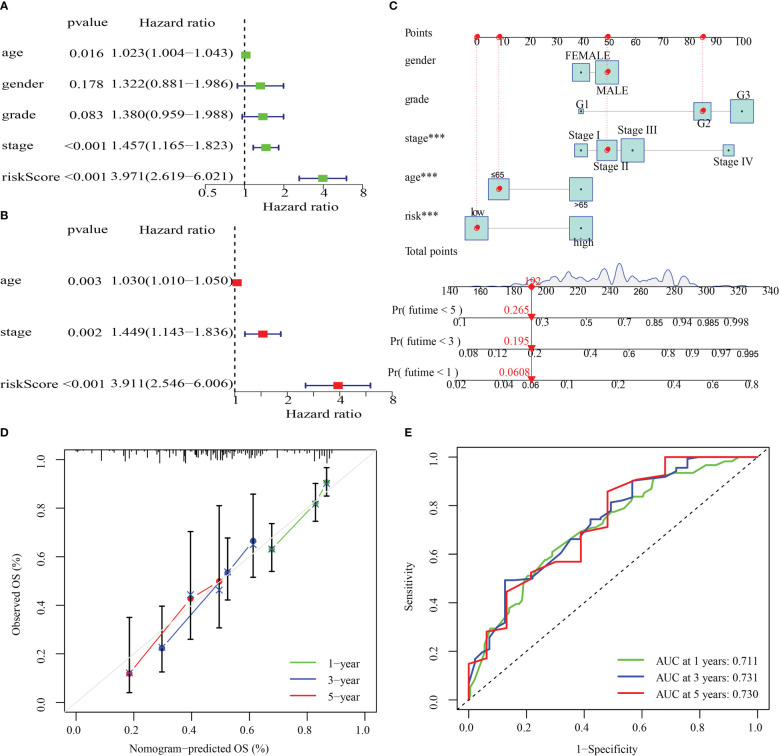
The development and validation of a risk score-based nomogram. **(A, B)**. Cox regression analysis, both univariate and multivariate, were performed on the training cohort. **(D)** Calibration curves for validating the nomogram’s capacity to predict outcomes over 1, 3, and 5 years. **(E)** The ROC curves’ AUC values offer a better assessment of the nomogram’s prognostic capacity. ***P < 0.001.

The clinical parameters (sex, grade, age, and stage) and risk score in the training cohort were then examined utilizing univariate and multivariate Cox regression analyses. By analyzing the risk score, stage, and age of the training cohort, we discovered that they independently functioned as prognostic indicators in both univariate and multivariate Cox regression models (**Figures 10A, B**). After that, a nomogram was produced by combining the aforementioned parameters (**Figure 10C**). Additionally, survival rates predicted by the nomogram and those observed were found to be in good agreement when calibration curves were established to verify the nomogram’s predictive capacity (**Figure 10D**). Values of the nomogram’s AUC were recorded to be 0.711, 0.731, and 0.730 over 1, 3, and 5 years, correspondingly (**Figure 10E**).

### 3.9 Verification of thirteen model genes by GEPIA and HPA platform

**Figure 11:**
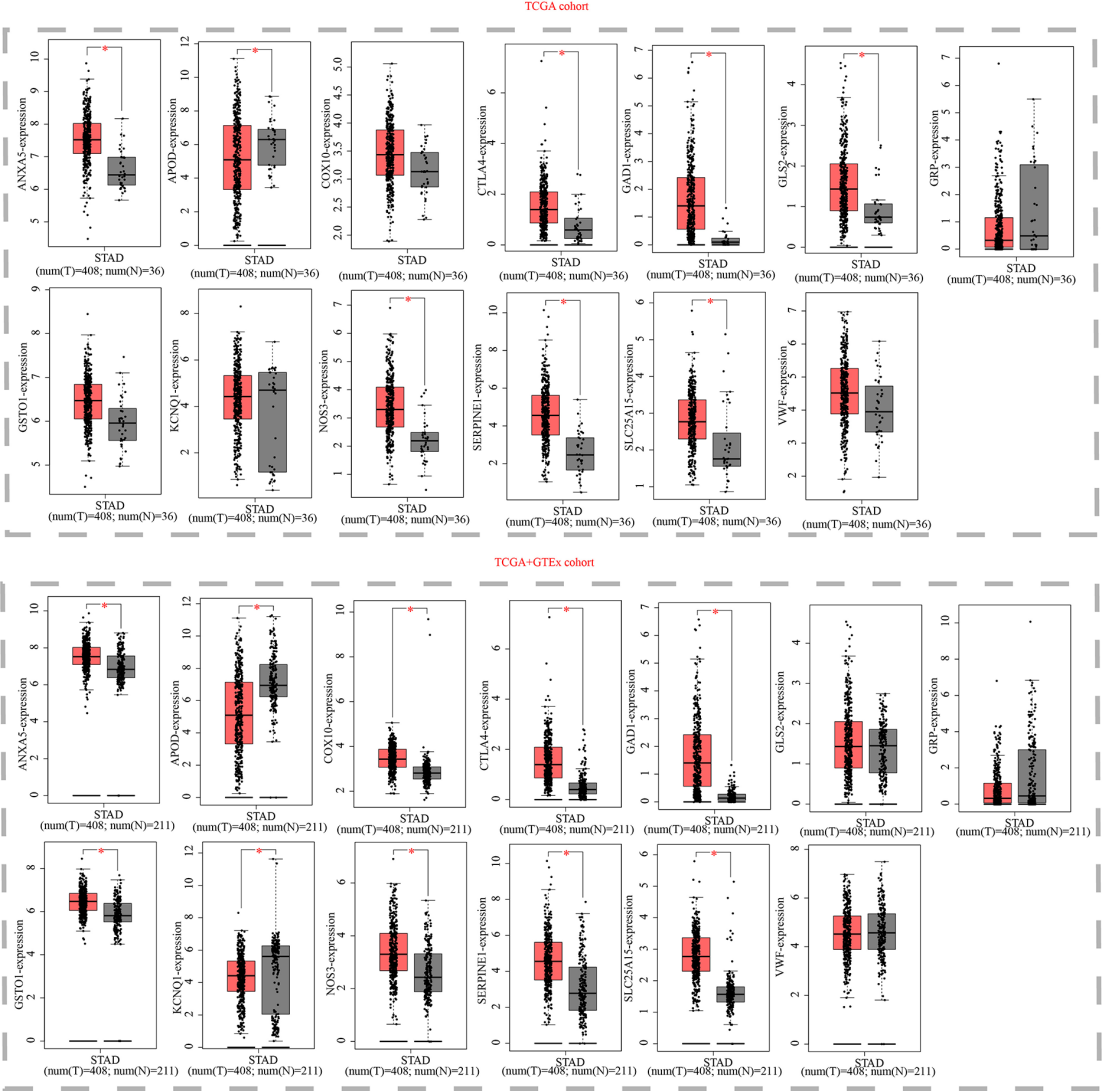
Expression validation of thirteen models OMRGs from the GEPIA database. (Box above represents the expression difference between STAD and normal samples derived from TCGA; the box below represents the expression difference between STAD and normal samples derived from TCGA and GTEX). *P < 0.05.

Finally, we examined the expression levels of the thirteen OMRGs using the GEPIA and HPA databases. The GEPIA database is a merger of the TCGA database and the GTEx database. The TCGA database offered all 408 of the tumor tissue samples for STAD, while the GTEx database gave 175 of the 211 normal gastric tissue samples and the TCGA database contributed 36. According to the GEPIA database, ANXA5, COX10, GAD1, GLS2, GSTO1, NOS3, SERPINE1, SLC25A15, VWF, and CTLA4 are all strongly expressed in STAD relative to normal tissue, whereas APOD, GRP, and KCNQ1 showed an opposite trend (**Figure 11**).

**Figure 12:**
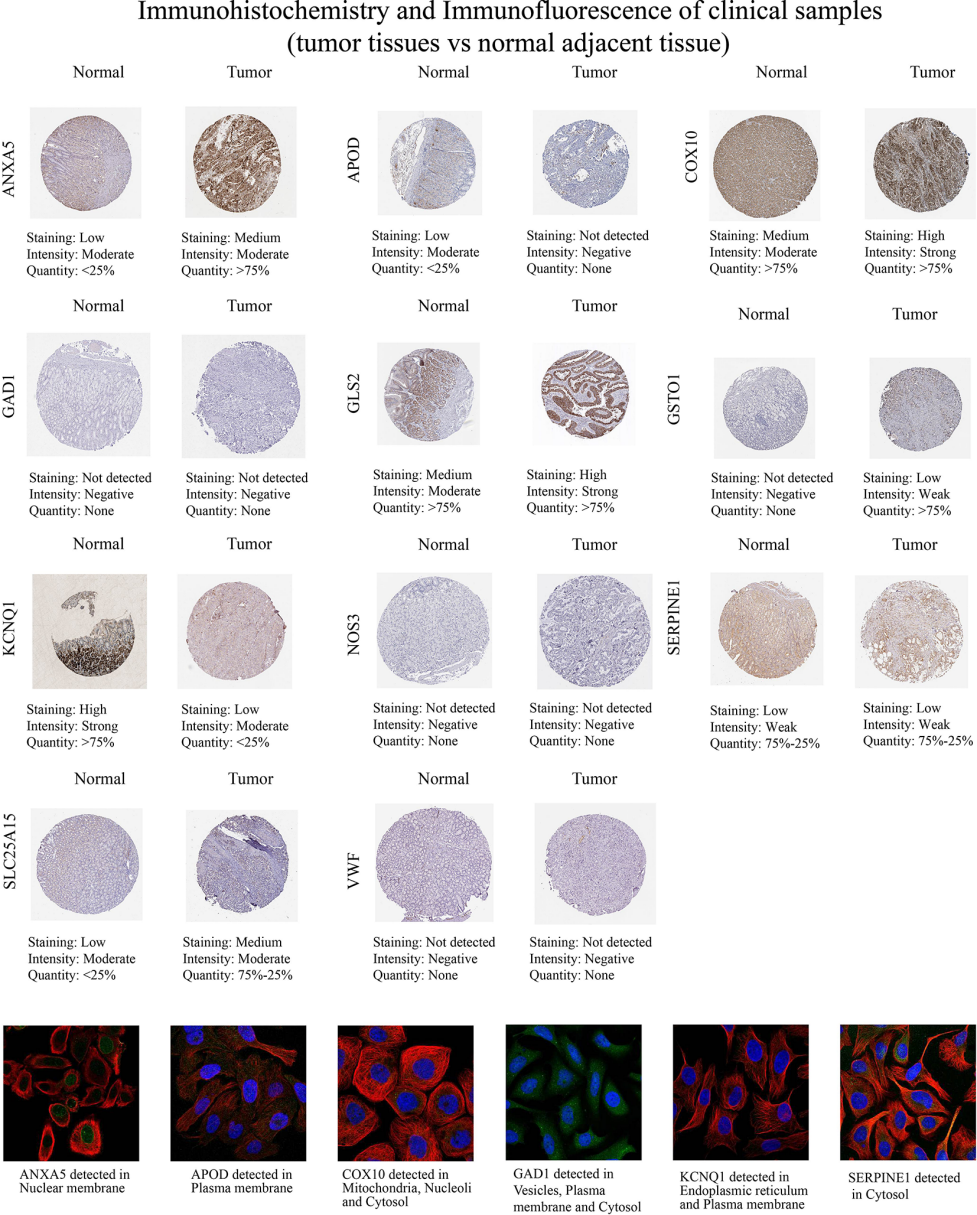
Immunohistochemistry and immunofluorescence of clinical samples (tumor tissues vs normal nearby tissues).

Then, we examined the immunohistochemistry (IHC) results of tumor and normal gastric tissues utilizing the HPA database to examine the levels of protein expressions of 13 genes (**Figure 12**). Because the HPA database did not provide information on the protein expression of CTLA4 and GRP, we performed the other 11 genes’ proteins. We found ANXA5, COX10, GLS2, GSTO1, and SLC25A15 protein levels were elevated in STAD as opposed to the normal tissues, while APOD and KCNQ1 were the opposite, which was in line with mRNA expression results from GEPIA. And GAD1, NOS3, SERPINE1, and VWF protein levels were not significantly different in tumor and normal samples. In addition, we explored the cellular localization of these genes, only ANXA5, APOD, COX10, GAD1, and KCNQ1 were found in the HPA database. The expression product of ANXA5, APOD, COX10, GAD1, and KCNQ1 was mainly located on the nuclear membrane, plasma membrane, mitochondria, nucleoli and cytosol, vesicles, plasma membrane and cytosol, and endoplasmic reticulum and plasma membrane, respectively (**Figure 12**).

## 4 Discussion

Oxidative stress has been linked to the onset and progression of cancer *via* diverse pathways, including the activation of cell survival, proliferation, and stress resistance systems. as well as enhancing genomic instability and mutagenicity. Meanwhile, the process of carcinogenesis is based on the reprogramming of cellular metabolism, which occurs either directly or indirectly as a result of carcinogenic mutations. For example, altered glucose metabolism is a hallmark of GC, and upregulated aerobic glycolysis in gastric cancer to meet the demands of cell proliferation is associated with genetic mutations, epigenetic modifications, and proteomic alterations (50). Likewise, abnormal lipid metabolism affects the development of GC. Low serum HDL levels have been found to predict higher risk of gastric cancer, higher rates of lymphovascular and vascular infiltration, advanced lymph node metastasis, and poor prognosis (51). In addition, dysregulated metabolism of amino acids has been identified as a metabolic regulator that supports cancer cell growth (52). Meanwhile, some studies have reported that the molecular pathways of oxidative stress are related to glucose metabolism or lipid metabolism (53). Within the context of cancer, the primary goals of PPPM are early detection, targeted prevention, prognosis, along with individualized management (54, 55). Even though a variety of treatments exists for STAD, including surgical intervention, immunotherapy, adjuvant chemotherapy, and endoscopic resection (56), the prognosis for patients with advanced STAD continues to be very dismal because there are no prognostic markers available for early diagnosis. Target therapy has emerged as a new therapeutic approach in recent years, and several tests have demonstrated its efficacy (57, 58). However, the molecular basis of STAD’s pathogenesis is still unknown. Thus, OMRG-based risk stratification of STAD is a promising strategy for prognosis assessment and personalized medicine.

As the study of oxidative metabolism has improved, researchers have uncovered the expanding roles that oxidative metabolism plays in the onset and advancement of cancer. Before looking at the effects of abnormal oxidative metabolism in STAD, we, therefore, describe the changes of OMRGs in a variety of malignancies. In actuality, partial OMRGs had predictive values for several malignancies, and OMRG variants occurred more or less frequently. In addition, the genetic mutations as well as the modifications of OMRGs were recognized in a variety of malignancies. OMRGs were positively linked to TNFA signaling *via* NFKB, KRAS signaling, inflammatory response, hypoxia, interferon Gamma response, and EMT in most types of tumors. Through its role in the regulation of these pathways, oxidative metabolism has been hypothesized to have a role in carcinogenesis.

After conducting further research into the connection between OMRGs and STAD, we clustered the samples into 3 clusters predicated on the scores assigned to their oxidative metabolism and the patterns of OMRG expression. The OS rates of patients whose oxidative metabolism was active were shown to be considerably lower compared to the rates of patients whose oxidative metabolism was inactive, indicating that the genes involved in oxidative metabolism were mostly risky. Considering that the intersecting metabolic reprogramming of tumor and immune cells is a potential mechanism *via* which antitumor immune response occurs in cancer, we additionally elucidated the link between OMRGs and immunological function, which could serve as a conceptual foundation for STAD immunotherapy in the long run. To estimate the stromal and immune composition of each patient, the ImmuneScore, StromalScore, and ESTIMATEScore were computed. TME is a niche made up of stromal cells, chemokines, and cytokines that support tumor tissues (59).

Greater ImmuneScore and StromalScore values correspond to greater TME components, respectively. These findings reveal that C2 subtypes linked to a worse outcome have a more robust immune abundance. In addition, the majority of immune-infiltrating agents were shown to have a positive correlation with OMRGs; this was especially true of macrophages, CCR, mast cells, type II IFN response, DCs, and T helper cells. The C2 subgroup’s prognosis was worse when there was a higher proportion of immunological components, indicating that immune checkpoint pathways were active. In three clusters, ICGs are subjected to differential expression. We discovered that ICGs are highly expressed in the C2 subtype, and these differentially expressed ICGs may be intrinsic to the differential prognosis of STAD and may be potential targets for treatment.

Currently, the first line of therapy for patients with advanced STAD is targeted drug therapy, however, its efficacy remains unsatisfactory and there is a need to identify a method to better predict the response to targeted drugs in STAD patients. Therefore, we explored whether there were discrepancies in the sensitivity of patients with three subtypes based on OMRG to commonly used chemotherapeutic agents. We discovered that the three patient groups had various medication sensitivity profiles, indicating that patients’ OMRG expression profiles might be used to tailor their treatment plans. For example, the use of Sunitinib, Dasatinib, Imatinib, Midostaurin, Bexarotene, and Pazopanib could be effective in treating patients whose oxidative metabolism is highly active, whereas Sorafenib, Paclitaxel, Methotrexate, Tipifarnib, and Vinorelbine could be effective in treating patients whose oxidative metabolism is inactive.

After that, to achieve an optimal signature that has clinical relevance, we screened 22 OMRGs by conducting a LASSO-cox regression analysis and assessed the optimal putative genes for signature creation. Following the completion of the validation, a novel OMRG-related prognostic signature that is comprised of 13 genes was developed (i.e., SLC25A15, GSTO1, VWF, ANXA5, SERPINE1, GRP, COX10, APOD, GAD1, NOS3, CTLA4, KCNQ1, and GLS2). Using the signature, STAD patients could be classified into the high-risk category, with a dismal prognosis, and the low-risk category, which has a favorable prognosis, in the train, test1, test2, and test3 cohorts. The AUC values of ROC curves demonstrated that the signature in question had an outstanding predictive performance. We investigated the difference in ICGs and immune function that exists between high- and low-risk categories of STAD patients because of the possible effect that the immune function and ICGs of the tumor might have on the treatment of the tumor. ICGs exhibit varied expressions in both groups. The upregulation of YTHDF1, CD160, TNFRSF25, CTLA4, TNFRSF14, JAK2, and CD244 and knockdown of TNFSF4, NRP1, CD276, and CD244 could be viable targets in STAD. Meanwhile, mast cells, MHC class I, parainflammation, Type II IFN response, and Th2 cells were statistically different in the high- and low-risk categories To make the most of the signature’s capacity for prediction, a nomogram was designed using the risk score and several other clinical data, and then a quantitative analysis was performed on the survival rate of patients suffering from STAD. Evaluation of the nomogram’s capacity to predict with a high degree of accuracy was carried out by means of calibration curves and ROC curves. Finally, we validated the thirteen model genes against the GEPIA and HPA databases.

There are some flaws in our research as well. First off, we only used retrospective data from the GEO and TCGA databases to validate the OMRG-based signature; going forward, we should conduct more prospective studies to assess its therapeutic implications. Meanwhile, additional sizable prospective clinical trials are required to evaluate its efficacy and usefulness.

## 5 Conclusion

In this study, patients with STAD could be classified into three clusters with different prognoses, immune characteristics, and drug sensitivity premised on OMRG scores. For the first time, an OMRG-related signature was developed and confirmed to accurately predict the prognosis of STAD patients. After that, utilizing this signature as well as other clinical parameters, a nomogram was generated as a quantitative tool to assist in predicting the survival rate for STAD patients. In summary, the present research has the potential to assist in the identification of prognostic predictions, targeted prevention, and individualized treatments for patients, hence proposing a new route to enhance PPPM for STAD.

## Data availability statement

The original contributions presented in the study are included in the article/**Supplementary Material**. Further inquiries can be directed to the corresponding authors.

## Author contributions

YD, QY, and JR contributed to this article equally. The content and authorship of the manuscript are entirely the responsibility of all authors. The study’s design, data collection and analysis, article preparation, and manuscript revision all benefited greatly from the efforts of all authors. All authors contributed to the article and approved the submitted version.
