# Supplementary material for: Identification and characterization of a novel molecular classification incorporating oxidative stress and metabolism-related genes for stomach adenocarcinoma in the framework of predictive, preventive, and personalized medicine
Source: Front Endocrinol (Lausanne). 2023 Feb 13;14:1090906. doi: 10.3389/fendo.2023.1090906 (PMC9969989; doi:10.3389/fendo.2023.1090906)
Supplement: Supplementary file 1 [file DataSheet_1.docx]

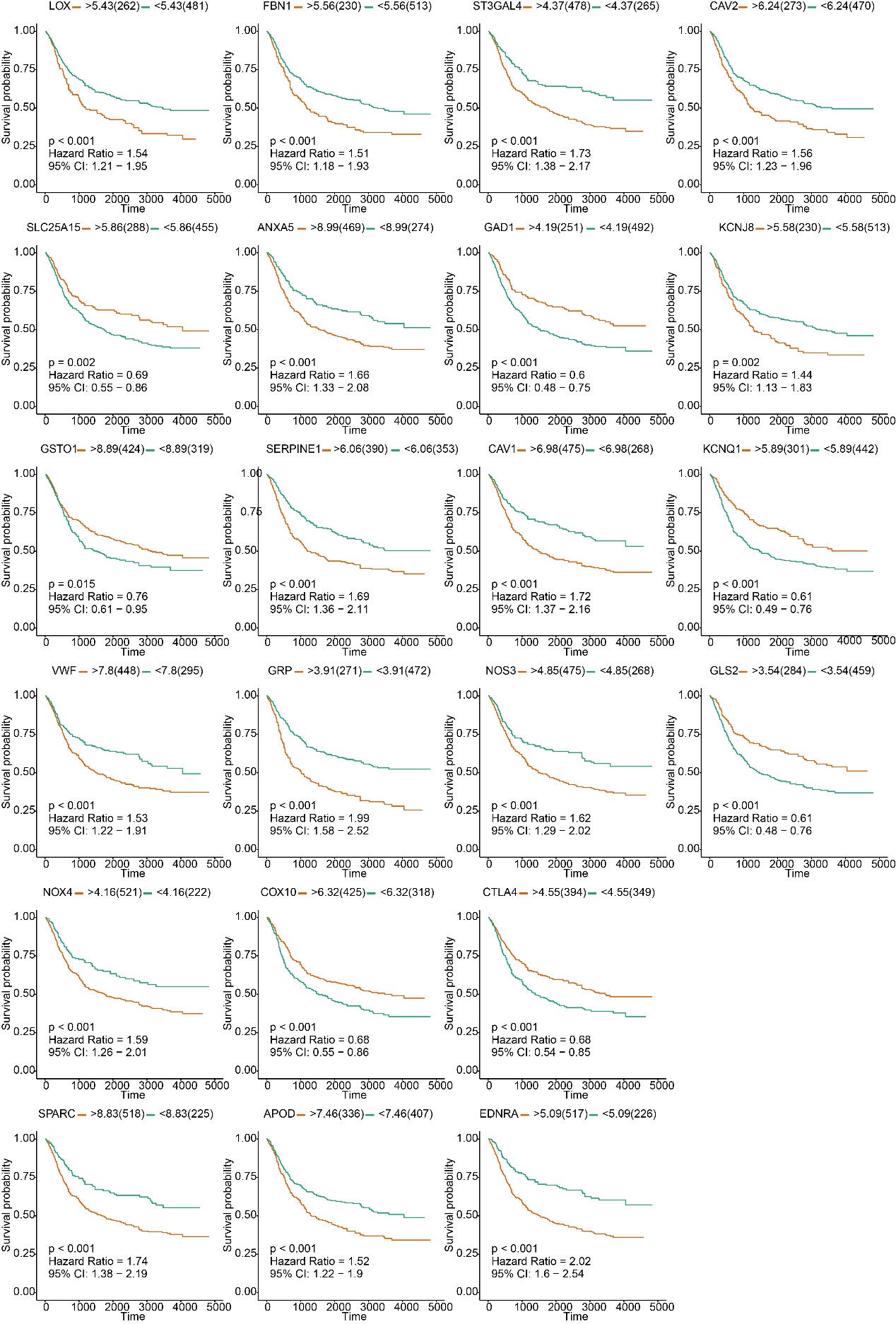


Supplementary Figure 1. The Kaplan-Meier curve showed that 22 OMRGs were prognostic relevant in STAD.


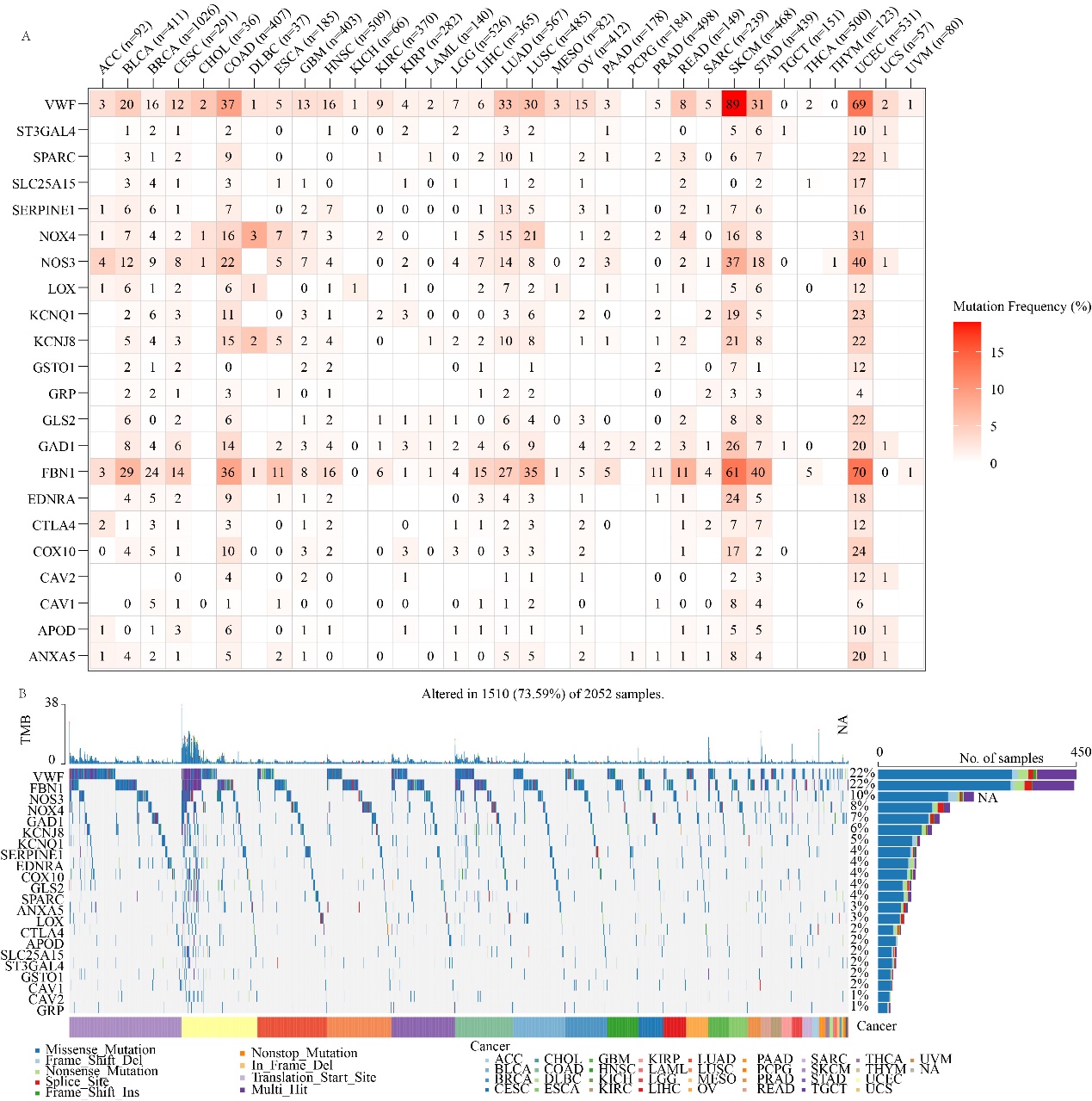


Supplementary Figure 2. Frequency of single nucleotide variations (SNVs) and various OMRG variant categories. (A) Mutation frequency of OMRGs. The numbers show how many samples of a certain tumor have the matching mutant gene. "0" denotes the absence of any mutation in the gene's coding region, while no number denotes the absence of any mutation in the gene at all. (B) SNV oncoplot. An oncoplot displaying the distribution of OMRG mutations and a list of SNV kinds.


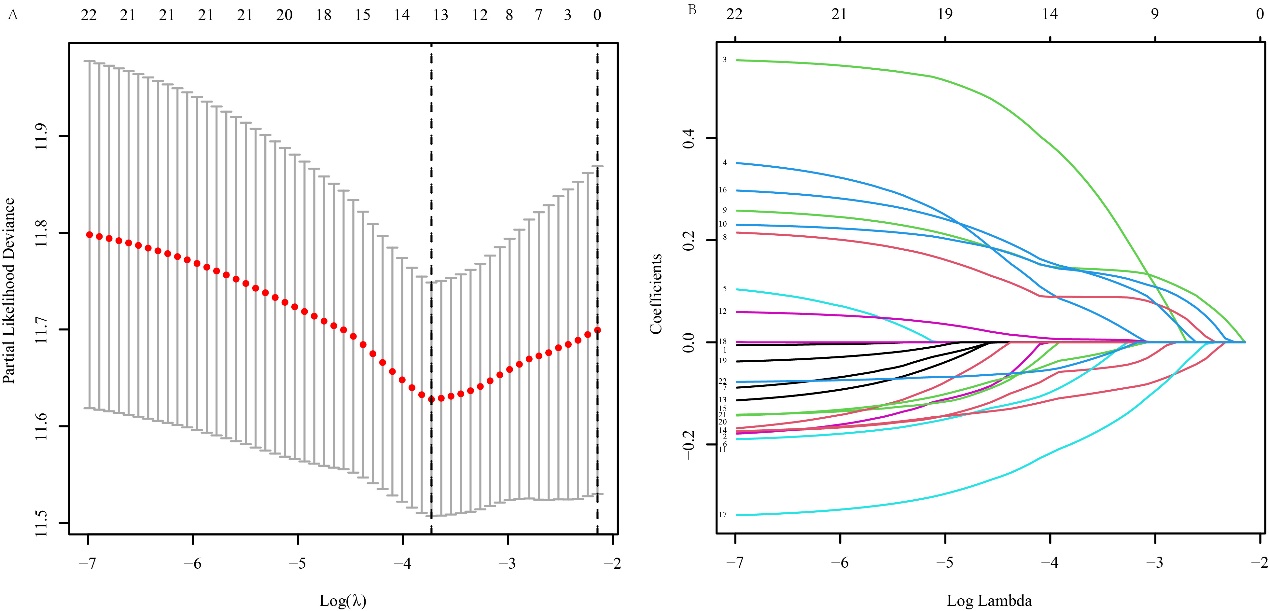


Supplementary Figure 3. Variable selection. (A-B) The LASSO-Cox regression analysis resulted in the selection of thirteen genes.


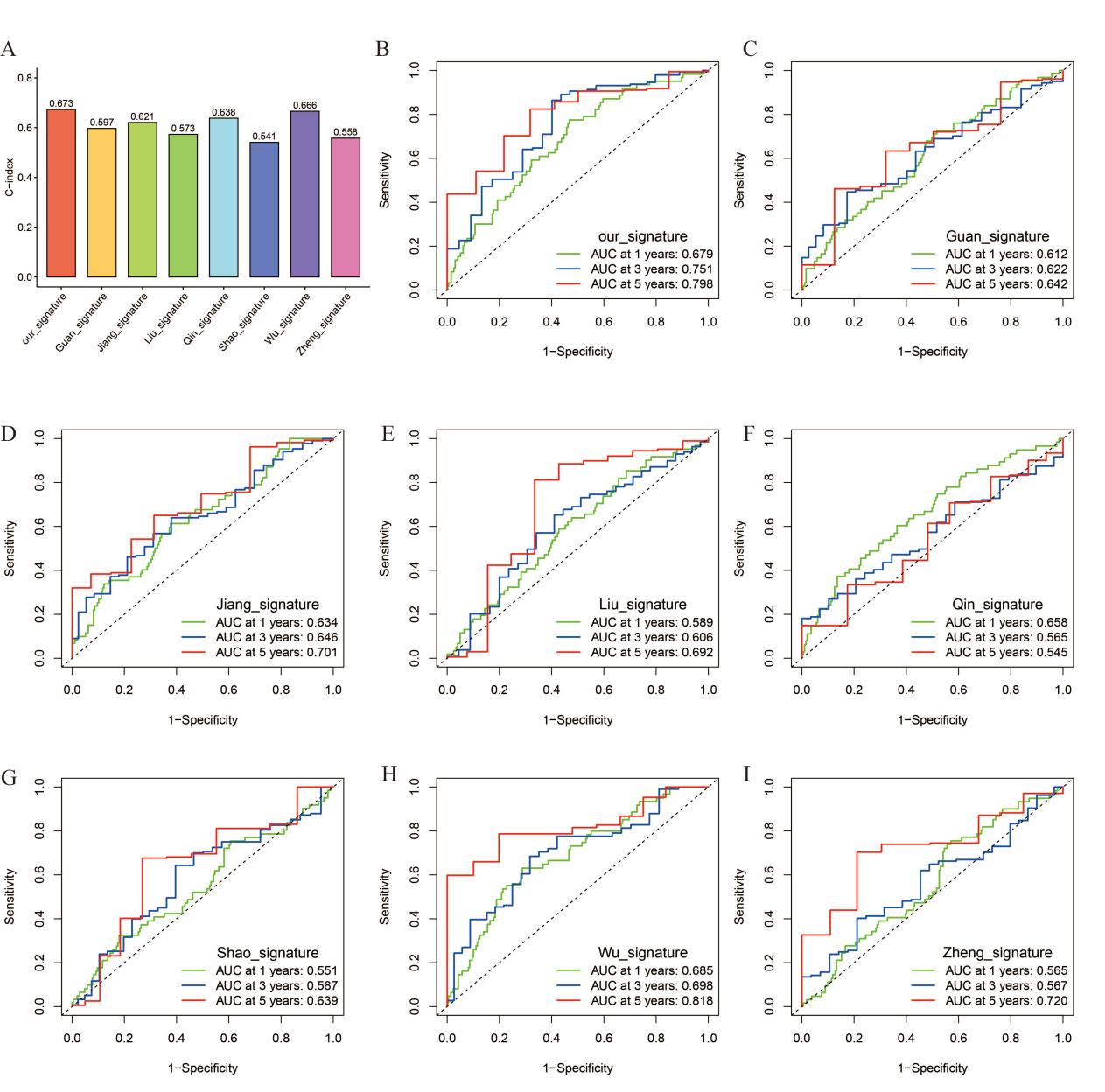


Supplementary Figure 4. Compare the accuracy of our signature with other well-established signatures. (A) The results of C-index. (B-I) The results of ROC curves of our signature, Guan signature, Jiang signature, Liu signature, Qin signature, Shao signature, Wu signature, and Zheng signature.
